# Erbliche neuroendokrine Neoplasien

**DOI:** 10.1007/s00292-026-01572-9

**Published:** 2026-05-28

**Authors:** Aziz Chouchane, Ilaria Marinoni, Aurel Perren

**Affiliations:** Institut für Gewebemedizin und Pathologie, Universität Bern, Murtenstrasse 31, 3008 Bern, Schweiz

**Keywords:** Keimbahnmutation, Hyperplasie, Protoonkogene, Tumorsuppressorgene, Multiple endokrine Neoplasie, Germ-line mutation, Hyperplasia, Proto-oncogenes, Tumor suppressor genes, Multiple endocrine neoplasia

## Abstract

Hereditäre neuroendokrine Neoplasien (NEN) sind die Grundlage von über 10 % aller neuroendokrinen Tumoren. Sie resultieren aus Keimbahnmutationen in Tumorsuppressorgenen oder Protoonkogenen.

Im Vergleich zu sporadischen Formen manifestieren sich hereditäre NEN meist im jüngeren Lebensalter. Charakteristisch sind eine multifokale Tumorentwicklung innerhalb eines Organs und das synchrone Auftreten in verschiedenen endokrinen Geweben. Histologisch sind spezifische Vorläuferläsionen wie endokrine Hyperplasien oder Mikrotumoren hinweisend. Immunhistochemische Untersuchungen können den Verlust von Genprodukten (z. B. SDHB, Menin) nachweisen und dienen als Wegweiser für die obligate molekulargenetische Keimbahnanalyse.

In dieser Übersicht werden klinische und histologische Hinweise erläutert, die auf einen familiären Hintergrund hinweisen können.

## Abstract (en)

Hereditary neuroendocrine neoplasms (NENs) are the basis of over 10% of all neuroendocrine tumors. They result from germline mutations in tumor suppressor genes or proto-oncogenes.

Compared to sporadic forms, hereditary NENs usually manifest at a younger age. Characteristic features include multifocal tumor development within a single organ or the synchronous occurrence of tumors in different endocrine tissues. Histologically, specific precursor lesions, such as endocrine hyperplasia or microtumors, can provide diagnostic clues. Furthermore, immunohistochemical analyses can directly detect the loss of gene products (e.g., SDHB, menin), serving as a guide for the mandatory molecular genetic germline analysis.

This overview explains the clinical and histological signs that may indicate a familial background.

Hereditäre neuroendokrine Neoplasien (NEN) sind eine klinisch und genetisch heterogene Gruppe, die etwa 10 % aller endokrinen Tumoren umfasst. Sie basieren auf Keimbahnmutationen in Tumorsuppressorgenen oder Protoonkogenen. Für Pathologen ist die Identifikation dieser Syndrome entscheidend, da die histologische Diagnose oft den ersten Hinweis auf eine familiäre Belastung liefert. Über 30 % der Phäochromozytome/Paragangliome (PPGL) [26], 25 % der medullären Schilddrüsenkarzinome (MTC) [17] 10–15 % der pankreatischen neuroendokrinen Tumoren (PanNET) [24], 10 % des primären Hyperparathyreoidismus und ein geringerer Anteil der verbleibenden neuroendokrinen Tumoren (NET) treten mit familiärem Hintergrund auf.

Interessanterweise sind bei bis zu 10 % der neuroendokrinen Karzinome (NEC) genetische Syndrome nachgewiesen, jedoch in klassischen Tumorsuppressorgenen von Tumordispositionssyndromen [28]. Auch ileale NET treten in familiärer Form auf, hier scheint jedoch jede Familie ihre eigene Keimbahnmutation aufzuweisen [8]. Hereditäre NEN manifestieren sich im Vergleich zu sporadischen Formen meist im jüngeren Lebensalter. Charakteristisch ist eine multifokale Tumorentwicklung innerhalb eines Organs oder das synchrone Auftreten von Tumoren in verschiedenen endokrinen Geweben. Mikroskopisch sind spezifische Vorläuferläsionen wie Hyperplasien endokriner Zellen oder Mikrotumoren histomorphologisch und immunhistochemisch erkennbar. Immunhistochemische Zusatzuntersuchungen können den Verlust von Genprodukten (z. B. SDHB, Menin, p27) nachweisen, was ein zusätzlicher Hinweis auf ein entsprechendes Syndrom sein kann. Analoge Mutationen können jedoch in unterschiedlicher Häufigkeit auch bei sporadischen Tumoren auftreten. Diese morphologischen und immunhistochemischen Befunde dienen als wichtige Wegweiser für die genetische Aufklärung. Falls der Patient eine anschließende molekulargenetische Keimbahnanalyse wünscht, kann die Diagnose damit gesichert werden. Eine Keimbahnanalyse ist auch die Grundlage eines Familienscreenings. In dieser Übersicht wird ein besonderes Augenmerk daraufgelegt, welche Aufgaben der Pathologie zukommen.

## Familiäre endokrine Tumorsyndrome

Bei den klassischen familiären endokrinen Tumorsyndromen stehen Tumoren endokriner Organe im Vordergrund. Nur selten treten anderweitige Tumoren auf (Tab. 1).

**Tab. 1:** Endokrine Tumorsyndrome

	Prävalenz/100.000	Endokriner Phänotyp	Histologie	Immunhistochemie
MEN1 *MENIN*	3–20	Nebenschilddrüsenadenome Hypophysenadenome NET des Pankreas Gastrinome des Duodenums NET von Magen, Lunge, Thymus	Multiglandulär Mikroadenome Multifokal Multifokal	MEN1-Verlust
MEN2 *RET*	3	MTC PCC Nebenschilddrüsenadenome	Multifokal Mikro-PCC	Keine
MEN4 *CDKN1B*	Sehr selten	Nebenschilddrüsenadenome Hypophysenadenome	–	P27-Verlust/Überexpression
MEN5 *MAXX*	Sehr selten	PPGL Hypophysenadenome	–	Unklar
HRPT2 *CDC73*	Sehr selten	Nebenschilddrüsenadenome und Karzinome	–	Verlust (nukleolären) Parafibromins
SDHx	2	PPGL, multifokal, maligne bei SDHB	Pseudohypoxie (Inh, CAIX)	SDHB-Verlust

*NET* neuroendokrine Tumoren, *MTC* medulläres Schilddrüsenkarzinom, *PPGL* Phäochromozytome und Paragangliome, *PCC* Phäochromozytom, *SDHB* Succinat-Dehydrogenase Untereinheit B

## Multiple endokrine Neoplasien

### Multiple endokrine Neoplasie Typ 1 (MEN1)

**Abb. 1:**
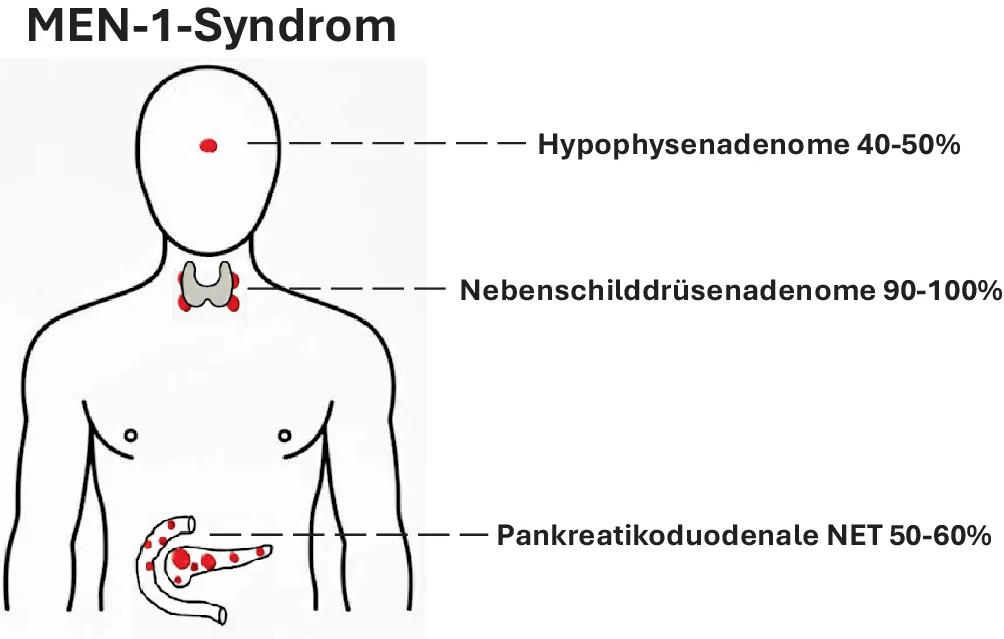
Phänotyp MEN-1-Syndrom

**Abb. 2:**
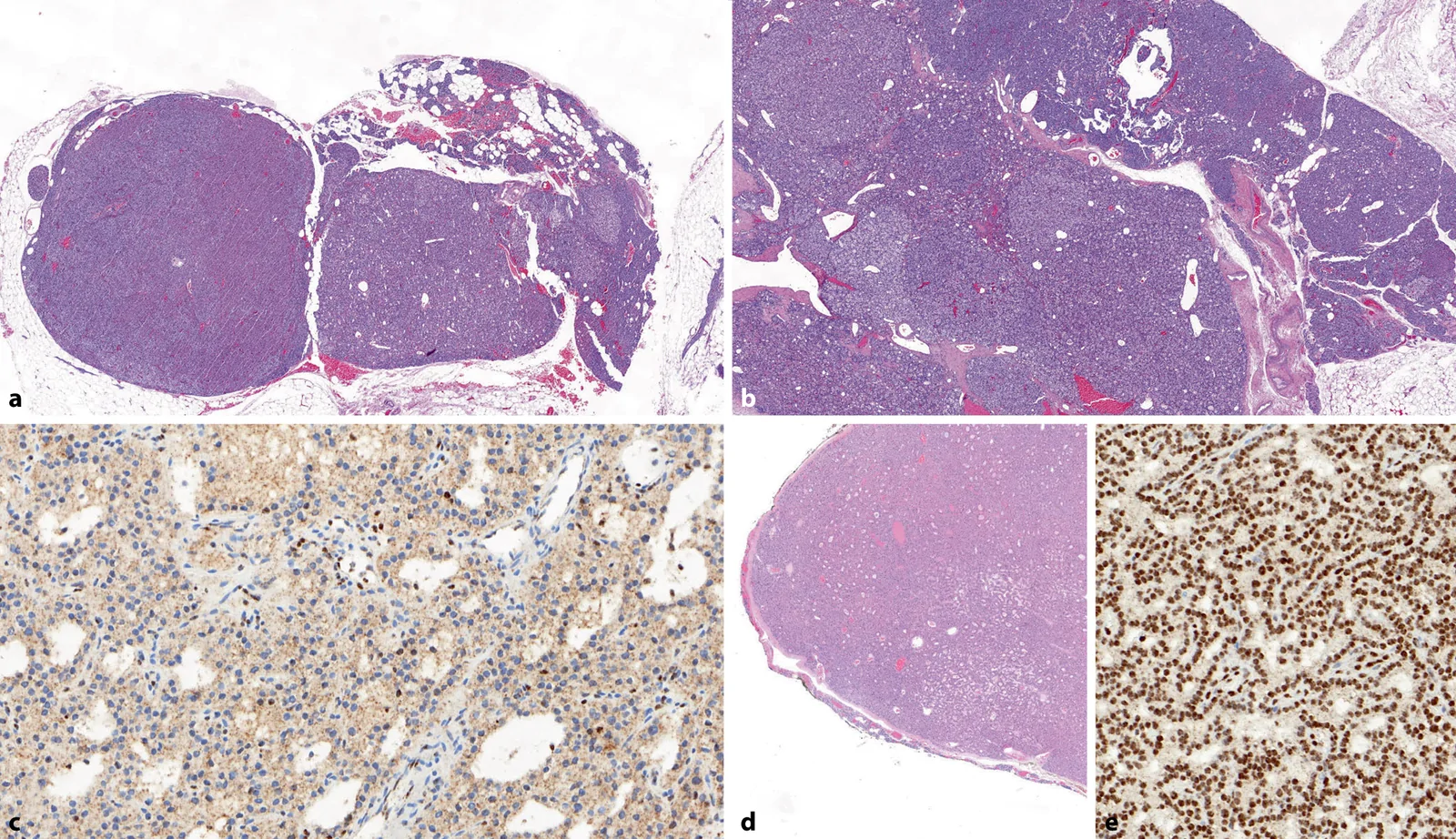
Nebenschilddrüsenadenome bei MEN1. **a**, **b** Nebenschilddrüsenadenome bei MEN1-Patienten mit Multinodularität, welche unabhängigen (sub-)klonalen Adenomen entsprechen. Es kann normales fettreiches Nebenschilddrüsengewebe wie bei **a** erhalten sein (HE, Originalvergr. 2,5:1). **c** Nukleärer Meninverlust in einem MEN1-assoziierten Nebenschilddrüsenadenom (Menin-Immunhistochemie, Originalvergr. 25:1). Achtung: Kerne der Stromazellen bleiben als interne Kontrolle positiv. **d**, **e** Sporadisches Nebenschilddrüsenadenom ohne Meninmutation (**d** HE, Vergr. 2,5:1; **e** Menin-Immunhistochemie, Vergr. 25:1)

**Abb. 3:**
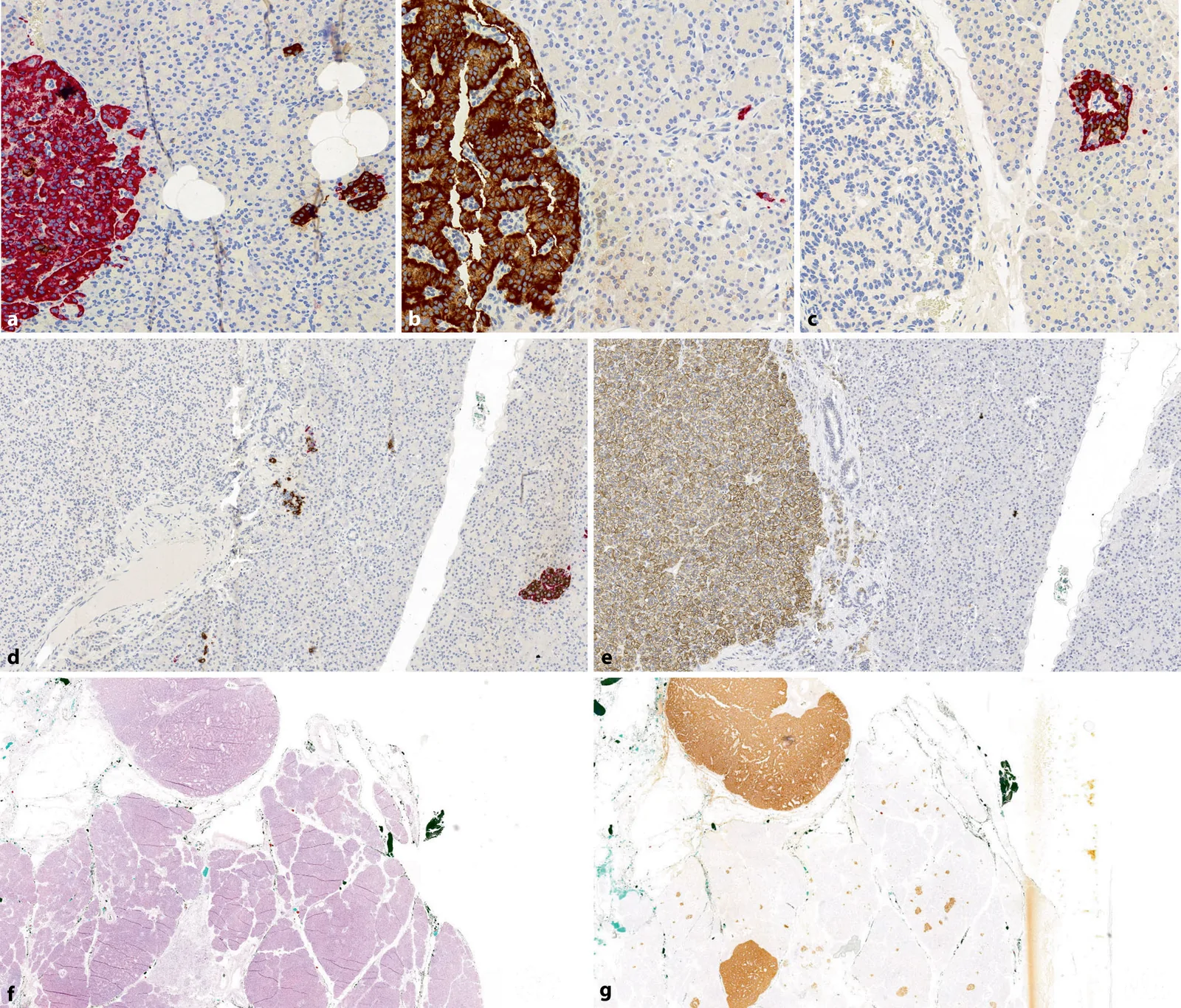
Mikroadenomatose. **a**–**c** Mikroadenomatose bei MEN1-Syndrom mit der Haupteigenschaft der Multihormonalität, Doppel-Immunhistochemie, Glukagon *rot* und Insulin *braun*, Originalvergr. 20:1. **a** Die meisten Mikroadenome exprimieren Glukagon (*rot*), normale Insel *rechts*. **b** Einzelne Mikroadenome können Insulin exprimieren (*braun*). **c** Einige Mikroadenome exprimieren andere oder keine Hormone (negativ für Insulin und Glukagon). **d**, **e** Mikroadenomatose bei Von-Hippel-Lindau-Syndrom. **d** Alle Mikroadenome sind negative für Hormonfärbungen (Doppel-Immunhistochemie, Glukagon *rot* und Insulin *braun*, Originalvergr. 10:1). **e** Mikroadenome weisen Pseudohypoxiemerkmale auf (MCT4, Originalvergr. 10:1). **f**, **g** Mikroadenome bei Insulinomatose: Alle sind positiv für Insulin. **f** HE, Originalvergr. 10:1. **g** Insulin, Originalvergr. 10:1

Klinisch steht beim MEN1-Syndrom meist ein primärer Hyperparathyreoidismus im Vordergrund, gefolgt von endokrinen Tumoren der Hypophyse, des Pankreas und Duodenums sowie seltener in Magen, Lunge und Thymus (Abb. 1; Tab. 1). Das MEN1-Syndrom wird durch inaktivierende Mutationen im *MEN1*-Gen auf Chromosom 11q13.1 verursacht, welches das Protein Menin codiert [5]. Der Hyperparathyreoidismus wird durch multiple (Mikro‑)Tumoren in den Nebenschilddrüsen verursacht [9]. Aufgrund des multiglandulären Auftretens wurde diese Erkrankung in der Vergangenheit als „primäre Hyperplasie der Nebenschilddrüse“ bezeichnet. Molekulare Untersuchungen haben jedoch bewiesen, dass diese vergrößerten Nebenschilddrüsen aus multiplen klonalen und subklonalen Tumoren bestehen [3], sodass es sich um multiple Adenoma handelt. MEN1-assoziierte Nebenschilddrüsenadenome sind histomorphologisch häufiger aus multiplen Knoten aufgebaut als sporadische Adenome und weisen fast immer einen immunhistochemischen Verlust von Menin auf (Abb. 2). In Duodenum und Pankreas ist das MEN1-Syndrom durch das Auftreten zahlreicher Vorläuferläsionen charakterisiert. Die Mikroadenomatose im Pankreas besteht aus multiplen Läsionen von der Größe einer Insel bis zu 5 mm. Diese endokrinen Mikrotumoren haben bereits eine klonale Inaktivierung des Wildtyp-*MEN1*-Allels [23]. Diese Inaktivierung resultiert in einem vollständigen Verlust der Menin-Expression, was immunhistochemisch nachgewiesen werden kann [12]. Interessanterweise exprimieren MEN1-assoziierte Mikrotumoren am häufigsten Glukagon, seltener Insulin oder andere pankreatische Hormone ([2]; s. Tab. 3; Abb. 3). Die Expression von nur einem Hormon und somit der Verlust der normalen multihormonalen Organisation der Inseln kann immunhistochemisch genutzt werden, um kleine Mikroadenome von Inseln zu unterscheiden. Insbesondere PP-Inseln (pankreatisches Polypeptid produzierende Inseln) im Processus uncinatus haben eine trabekuläre Architektur und sollten nicht mit Mikroadenomen verwechselt werden.

MEN1-assoziierte Gastrinome treten vor allem im Duodenum auf. Die Primärtumoren sind häufig sehr klein und multifokal. Die daneben vorliegende Hyperplasie der G‑Zellen ist schwer erkennbar [1]. Häufig sind die Lymphknotenmetastasen, die teils peripankreatisch liegen, deutlich größer als die Primärtumoren. Aus diesem Grund wurde wahrscheinlich bei den sog. Lymphknotengastrinomen der mikroskopische Primärtumor im Duodenum nicht detektiert. Klinisch sollte bei jedem Gastrinom im Duodenum (Zollinger-Ellison-Syndrom) stets eine MEN1-Assoziation geprüft werden, da diese Tumoren dann multipel auftreten und die Therapie anders als bei sporadischen Gastrinomen geplant wird. Im Gegensatz zu den Gastrinomen mit ZES-Syndrom sind der Großteil der MEN1-assoziierten PanNET klinisch nichtfunktionell. Bei funktionell aktiven PanNET ist aufgrund des multifokalen Auftretens von pankreatischen und duodenalen NET bei MEN1-Patienten eine immunhistochemische Bestätigung der Hormonexpression in resezierten Tumoren wichtig. So lässt sich beweisen, dass der resezierte Tumor Ursprung des Syndroms war. Interessanterweise können MEN1-assoziierte Insulinome im Verlauf eine ektope GHRH-Produktion erlangen [15]. Im Verlaufe der Tumorprogression weisen MEN1-assoziierte PanNET häufiger Mutationen in den Chromatin-modifizierenden Genen *DAXX* oder *ATRX* auf. Dies manifestiert sich immunhistochemisch durch einen nukleären Verlust der entsprechenden Proteine bei höhergradigen Tumoren (G2/G3) und geht mit einem erhöhten Risiko für Metastasen einher [7].

Die MEN1-assoziierten Hypophysenadenome sind meist hormonaktiv, zeigen jedoch keine spezifischen histologischen Merkmale, die sie von sporadischen Adenomen unterscheiden, abgesehen von einer tendenziell höheren Aggressivität und immunhistochemisch plurihormonaler Expression.

### Multiple endokrine Neoplasie Typ 2 (MEN2)

**Abb. 4:**
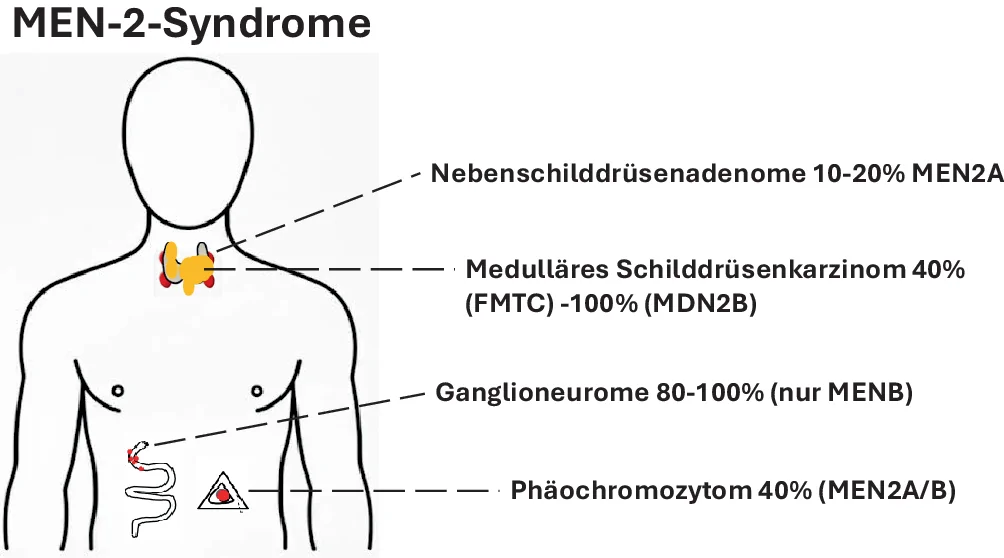
Phänotyp MEN-2-Syndrom

Das MEN2-Syndrom resultiert aus aktivierenden Keimbahnmutationen im *RET*-Protoonkogen auf Chromosom 10q11.2 [20]. Leittumorerkrankung ist das medulläre Schilddrüsenkarzinom (MTC), das in allen Ausprägungsformen der Erkrankung und bei den meisten Anlageträgern auftritt (Abb. 4). Histomorphologisch unterscheiden sich familiäre MTC nicht von sporadischen MTC. Beide können typische Amyloidablagerungen aufweisen. MTC haben ein breites morphologisches Erscheinungsbild. Schon bei geringem morphologischem Verdacht auf ein MTC ist eine Calcitonin-Immunhistochemie indiziert. Ein Auftreten der Erkrankung in jungen Jahren und ein multifokales Auftreten sind histopathologische Hinweise auf eine familiäre Ursache. Da heutzutage allen Patienten eine Keimbahnanalyse angeboten wird, ist die Beschreibung einer assoziierten „C-Zell-Hyperplasie (CCH)“ nicht mehr zentral. CCH ist definiert als eine Vermehrung von C‑Zellen (> 50/Gesichtsfeld) mit einem intrafollikulären Wachstumsmuster ohne Durchbruch der Basalmembran. Die Unterscheidung zwischen CCH und medullärem Karzinom gestaltet sich manchmal schwierig, hier sind der Verlust eines follikelassoziierten Wachstums, eine desmoplastische Stromareaktion sowie klar infiltratives Wachstum zentral. Bei der Untersuchung eines MTC und einer CCH spielt die immunhistochemische Untersuchung für Calcitonin und CEA eine zentrale Rolle.

Phäochromozytome treten bei MEN2A und MEN2B in etwa 40 % der Fälle auf, sind häufig bilateral und gehen oft mit einer nodulären Nebennierenmarkhyperplasie einher, wobei diese Knoten molekular auch Mikrotumoren sind. Histologisch sind die Tumoren nicht von sporadischen Formen zu unterscheiden. MEN2-assoziierte Phäochromozytome produzieren Adrenalin und Noradrenalin und zeigen ein geringes Malignitätspotential [10]. Bei MEN2A tritt zusätzlich zu MTC und Phäochromozytomen auch Hyperparathyreoidismus auf. Dieser ist wie bei der MEN1 durch teils multifokale Adenome verursacht, welche früher ebenfalls fälschlicherweise als Hyperplasie bezeichnet wurden.

Bei der aggressivsten Variante, der MEN2B mit aktivierenden *RET*-Mutationen in der Kinasedomäne treten neben (frühkindlichen) MTC auch Ganglioneurome an der Zunge und im Gastrointestinaltrakt auf, welche auf ein MEN2-Syndrom hindeuten können.

### MEN4, MEN5

Das MEN2B-Syndrom wurde früher als MEN3 bezeichnet, daher wird dieser Begriff nicht mehr genutzt. In den letzten Jahren wurden das MEN4-Syndrom (Mutationen im *CDKN1B*-Gen, das das p27-Protein codiert) und MEN5-Syndrom beschrieben (Mutation in *MAX*). Beide ähneln klinisch und histomorphologisch der MEN1, sind jedoch viel seltener. MEN4-Patienten zeigen Hyperparathyreoidismus und Hyophysenadenome sowie selten NET. Ein immunhistochemischer p27-Proteinverlust ist nicht immer nachweisbar, da inaktivierende Mutationen auch zu p27-Akkumulation führen können [18]. MEN5-Patienten weisen (bilaterale) Phäochromozytome auf, seltener Hypophysenadenome. Die Wertigkeit eines immunhistochemischen Verlust des MAX-Proteins ist noch nicht geklärt [6].

### Hyperparathyroidism-jaw-tumor-Syndrom und familiärer Hyperparathyroidismus

Das Hyperparathyroidism-jaw-tumor-Syndrom (HRPT2) ist durch inaktivierende Mutationen des CDC73**-**Gens verursacht [4, 25]. Das Syndrom ist für weniger als 1 % der Nebenschilddrüsenadenome verantwortlich. Diese Adenome sind durch hohe Parathormonwerte und braune Tumoren klinisch charakterisiert. Bei Verdacht kann ein immunhistochemischer Verlust von Parafibromin, dem Genprodukt von *CDC73* in Adenomen auf das Syndrom hinweisen. Während sporadische Nebenschilddrüsenadenome keinen Parafibromin-Verlust aufweisen, ist in bis zur Hälfte der sporadischen Nebenschilddrüsenkarzinome Parafibromin verloren. Dieser Marker kann hier diagnostisch eingesetzt werden und lässt auf ein aggressives Verhalten von atypischen Nebenschilddrüsenadenomen schließen [13, 16].

Bei anderen Formen des hereditären Hyperparathyreoidismus einschließlich der familiären hypocalciurischen Hypercalcämie (FHH), des neonatalen primären schweren Hyperparathyreoidismus (NSHPT) sowie des isolierten familiären Hyperparathyreoidismus (FIHPT) hat der Pathologe zurzeit keine wichtige Rolle.

### Paragangliome und SDH-defiziente Tumorsyndrome

**Abb. 5:**
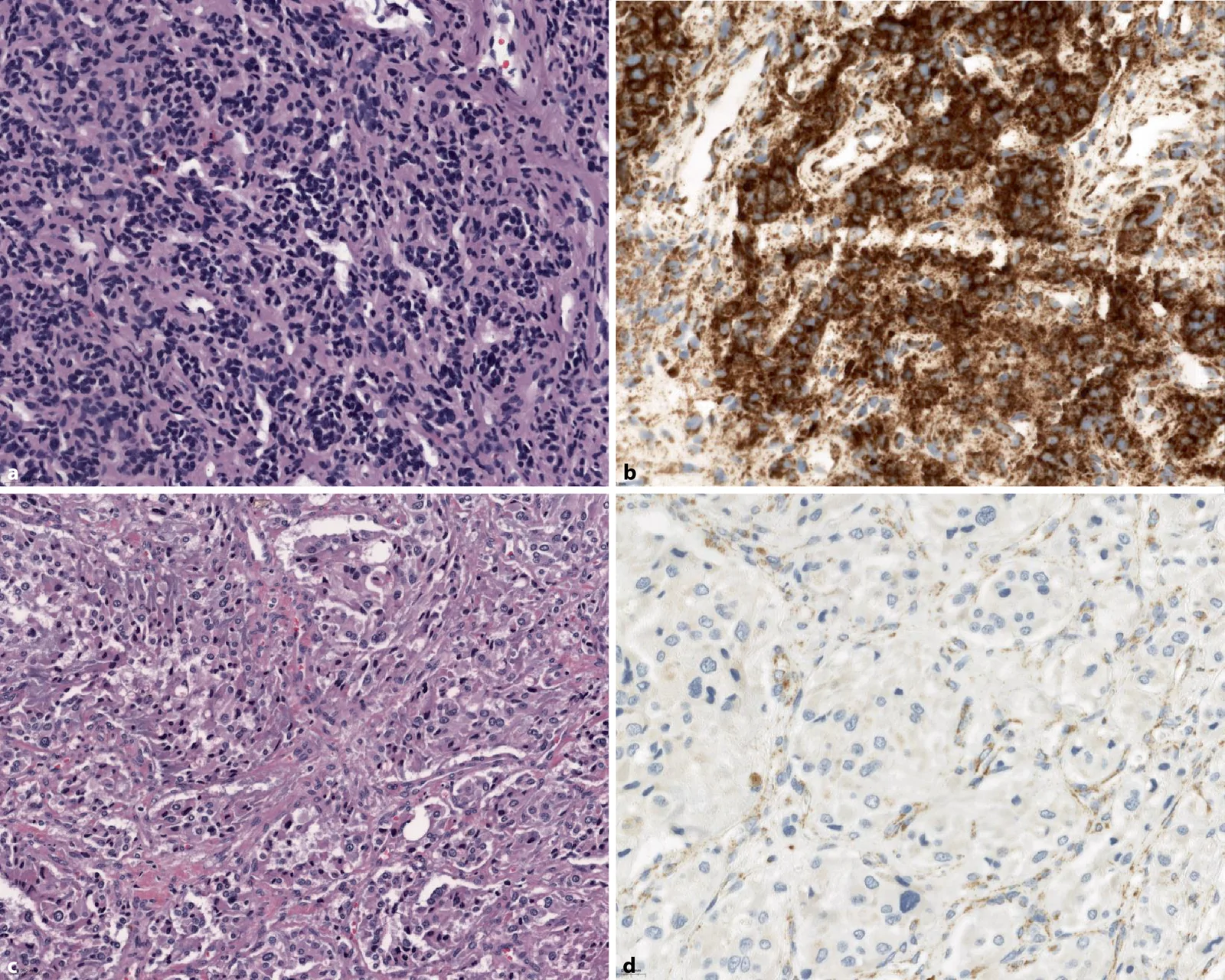
SDH-assoziierte Phäochromozytome/Paragangliome. **a** Hämatoxylin-Eosin-Färbung eines SDHB-Wildtyp-sympathischen Paraganglioms. **b** Die SDHB-immunhistochemische Färbung zeigt eine diffuse granulare zytoplasmatische Positivität. **c** Hämatoxylin-Eosin-Färbung eines SDHB-mutierten adrenalen Phäochromozytoms. **d** Die SDHB-immunhistochemische Färbung zeigt einen Verlust der granulären (mitochondrialen) Expression in den Tumorzellen (Endothelzellen als interne Positivkontrolle)

Über 30–40 % der Phäochromozytome und Paragangliome (PPGL) haben einen familiären Hintergrund [26]. Neben MEN2, VHL, NF1 sind Mutationen in den Genen des Succinat-Dehydrogenase-Komplexes (*SDHA, SDHB, SDHC, SDHD*) in PPGL häufig. Der SDH-Komplex spielt eine zentrale Rolle im Krebs-Zyklus und eine Inaktivierung führt zur Akkumulation des Onkometaboliten Succinat. Dies aktiviert über Abbau von Prolylhydroxylasen und HIF eine Hypoxiekaskade trotz Anwesenheit von genügend Sauerstoff, eine sog. Pseudohypoxie. *SDHx*-Keimbahnmutationen sind für einen großen Teil familiärer PPGL verantwortlich und können durch einen Verlust der granulären (Mitochondrialen) SDHB-Positivität identifiziert werden (Abb. 5). Dies widerspiegelt die Verhinderung der Bildung des mitochondrialen Komplex II der Respirationskette, sobald eines der SDH-Proteine defizient ist. Diagnostisch wichtig ist der Erhalt der Positivität in Endothelzellen als interne Positivkontrolle. Wenn zusätzlich die SDHA-, SDHC- oder SDHD-Färbung verlorengeht, weist dies spezifisch auf eine *SDHA*-/*SDHC-* oder *SDHD*-Mutation hin [27].

Die Multifokalität von familiären PPGL sollte nicht als Metastasierung fehlinterpretiert werden. PPGL können überall außer in Lymphknoten und im Knochen auftreten. Solange kein Rest-Lymphknoten mikroskopisch identifiziert werden kann, ist ein Zweittumor einer (Lymphknoten‑)Metastase vorzuziehen [26].

Molekular gehören *SDH*- und *VHL*-assoziierte Phäochromozytome zur Pseudohypoxiegruppe. Auf Proteinebene exprimieren sie die Hypoxiemarker α‑Inhibin und CAIX und sind mit einer hohen Mikrogefäßdichte assoziiert [26].

Keimbahnmutationen von *SDHB* nehmen eine besondere Rolle ein, da sie mit einem deutlich höheren Risiko für eine Metastasierung der Paragangliome assoziiert sind. Diese Patienten benötigen deshalb engmaschige klinische Verlaufskontrollen [10]. Keimbahnmutationen in *MAX, TMEM127, EGL1* und anderen Genen sind selten und es bestehen keine klaren morphomolekularen Korrelationen.

## Familiäre Tumorsyndrome mit endokrinen Neoplasien

Neben den Tumorsyndromen mit überwiegend endokrinen Neoplasien können auch bei nichtendokrinen Tumorsyndromen neuroendokrine Neoplasien einen Teil des Erscheinungsspektrums bilden. Die 3 Tumorsyndrome mit dem häufigsten Auftreten neuroendokriner Neoplasien werden separat aufgeführt. Es scheint jedoch bei sehr vielen Tumorsyndromen selten auch zur Entstehung neuroendokriner Neoplasien zu kommen. So wurden z. B. bei Keimbahnanalysen von unselektionierten NET und NEC-Patienten bei etwa 10 % der Patienten ein Tumorsyndrom gefunden, einschließlich BRCA2, APC, HNPCC, Li-Fraumeni [24, 28]. Molekular weisen die NEN dieser Patienten entsprechende Mutationssignaturen auf, sodass von einem kausalen Zusammenhang ausgegangen werden kann (Tab. 2).

**Tab. 2:** Tumorsyndrome mit neuroendokrinen Tumoren

	Häufigkeit von endokrinen Tumoren	Phänotyp	IHC
Von-Hippel-Lindau-Syndrom *VHL*	20 %	Nierenzellkarzinome Hämangioblastome *familiäres Paragangliom‑/Phäochromozytom-Syndrom* *NET des Pankreas*	Pseudohypoxie (Inhibin, CA IX, MCT4)
Neurofibromatose Typ 1 *NF1*	7 %	Café-au-lait-Flecken Neurofibrome *Phäochromozytome* *NET der Ampulla*	NF1-Verlust in einem Teil der Tumoren
Tuberöse Sklerose *TSC1*	Selten	Neurologische Symptome Angiomyolipome Lymphangioleiomyomatose *NET*	–
*BRCA* *ATM* *APC*	Selten	*NEC und NET*	–

*NEC* neuroendokrine Karzinome, *NET* neuroendokrine Tumoren

### VHL-Syndrom

Das Von-Hippel-Lindau-Syndrom resultiert aus Mutationen im *VHL*-Gen (3p25.3). Diese führen zu einer gestörten Regulation des Hypoxie-induzierten Faktors (HIF) mit molekularer Pseudohypoxie. Während bei VHL-Typ 1 (mit Deletionen und trunkierenden Mutationen) hellzellige Nierenzellkarzinome und Hämangioblastome führend sind, haben 50–80 % der VHL-Typ-2-Patienten mit Missense-Mutationen PPGL und seltener auch PanNET [19]. Diese Tumoren weisen, wie PPGL bei SDH-Mutationen, einen Pseudohypoxiephänotyp auf, der durch Positivität für α‑Inhibin und CAIX erkannt werden kann. Für diese PPGL und PanNEN bei VHL-Patienten besteht seit kurzem eine neu zugelassene, zielgerichtete Therapie gegen Hif2a (Belzutifan) [14]. PanNET bei VHL treten häufig multifokal, teils unter dem Bild einer Mikroadenomatose auf. Diesen Mikroadenomen (Mikrotumoren) fehlt die Hormonexpression und sie zeigen Pseudohypoxiemerkmale wie CAIX- oder MCT4-Expression (Tab. 3; [22]). Ein wichtiges diagnostisches Kriterium ist die Assoziation mit multiplen serösen Zystadenomen des Pankreas, die bei VHL-Erkrankung das ganze Pankreas ersetzen können.

**Tab. 3:** Differenzialdiagnose der Mikroadenomatose des Pankreas

Diagnose	Hormon-Expression der Mikrotumore	Inselhyperplasie	Andere
Multiple endokrine Neoplasie Typ 1	Multihormonal, am häufigsten Glukagon	Selten	Neuroendokrine Tumoren des Pankreas mit verschiedenen Hormonen
Von-Hippel-Lindau-Syndrom	Kein Hormon	Nein	Pseudohypoxie (CAIX) seröse mikrozystische Adenome
Glucagon-Zell-Adenomatose	Nur Glukagon	Überwiegend α‑Zell-Hyperplasie	Glukagon-produzierende neuroendokrine Tumore des Pankreas
(MAFA) Insulinomatose	Nur Insulin	Nein	Multiple Insulinome

### NF-Syndrom

Die Neurofibromatose Typ 1 (Recklinghausen-Krankheit) wird durch Mutationen im *NF1*-Gen verursacht. Neben Neurofibromen, Café-au-lait-Flecken und Gliomen treten bei ca. 10 % der Patienten duodenale Somatostatin-produzierende NET bevorzugt in der periampullären Region auf [11]. Ein hochspezifisches histologisches Merkmal, das dem Pathologen die Diagnose Somatostatin-produzierender Tumor nahelegt, ist das Vorkommen von Psammonkörperchen innerhalb des oft pseudoglandulär aufgebauten Tumors. Trotz der hormonellen Expression von Somatostatin präsentieren sich diese Patienten klinisch selten mit einem funktionellen Syndrom [11]. Phäochromozytome treten bei NF1 seltener auf als bei MEN2 oder VHL, zeigen aber das gleiche morphologische Bild. NF‑1 assoziierte PCC sind teils multifokal und häufig Composit Phäochromozytome/Ganglioneurome. Molekular gehören sie gemeinsam mit MEN2-PCC zur Kinase-Signalweg-Gruppe mit hoher Expression von PNMT, welches Noradrenalin zu Adrenalin metabolisiert. Diese Phäochromozytome haben ein geringeres Malignitätsrisiko.

### TSC1-Syndrom

Der Tuberöse-Sklerose-Komplex (TSC), verursacht durch Mutationen in *TSC1* oder *TSC2*, führt zu einer Überaktivierung des mTOR-Signalwegs. Leitneoplasien sind Angiomyolipome und low-grade muzinöse Neoplasien (LAMN) der Lunge [21]. Neuroendokrine Tumoren des Pankreas sind bei TSC eine seltene Manifestation. Die Tumoren sind meist gut differenziert, unifokal (G1 oder G2) und zeigen keine spezifische morphologische Abgrenzung zu sporadischen NEN. Diagnostisch wegweisend ist lediglich die Klinik und Assoziation zu anderen Neoplasien.

## Fazit für die Praxis

- Die pathologische Diagnose familiärer neuroendokriner Neoplasien erfordert eine hohe Aufmerksamkeit für klinischen Kontext und subtile morphologische Hinweise.
- Wesentliche Indikatoren für eine hereditäre Genese sind junges Alter der Patienten, die Multifokalität der Tumoren und das Vorliegen spezifischer Vorläuferläsionen wie die Mikroadenomatose im Pankreas, Mikro-Phäochromozytome oder die C‑Zell-Hyperplasie in der Schilddrüse.
- Immunhistochemische Algorithmen sind heute integraler Bestandteil der Befundung: Der routinemäßige Einsatz von SDHB-Färbungen bei allen Paragangliomen (PPGL) sowie die Untersuchung der Menin-Expression bei multiplen neuroendokrinen Neoplasien des Pankreas (PanNEN) sind essenziell. Indirekt über Marker der Pseudohypoxie können Hinweise auf eine VHL- oder SDH-assoziierte Erkrankung gewonnen werden.
- Bei PPGL ist immer eine Multifokalität auszuschließen, bevor eine (Lymphknoten‑)Metastase diagnostiziert wird. Die Befundung neuroendokriner Tumoren sollte stets zusätzlich zum Grading (Ki-67-Index) auch Hinweise zur Multifokalität enthalten, da dies der relevanteste Hinweis auf eine familiäre Genese ist.
- Da die korrekte Einordnung weitreichende Konsequenzen für das klinische Management und die Familienberatung hat, sollte bei jedem begründeten morphologischen Verdacht eine genetische Abklärung empfohlen werden.

## Data Availability

Alle dieser Arbeit zugrunde liegenden Daten sind in diesem Artikel enthalten.
